# Determinants of the health care service choices of long-term mechanical ventilation patients: Applying andersen’s behavioral model

**DOI:** 10.1371/journal.pone.0274303

**Published:** 2022-09-09

**Authors:** Hui-Yu Liang, Ming-Der Lee, Kuan-Chia Lin, Lieh-Hann Lin, Shu Yu

**Affiliations:** 1 School of Nursing, National Taipei University of Nursing and Health Sciences, Taipei, Taiwan; 2 Department of Long-Term Care, National Taipei University of Nursing and Health Sciences, Taipei, Taiwan; 3 Community Research Center, Institute of Hospital and Health Care Administration, National Yang Ming Chiao Tung University, Taipei, Taiwan; 4 Department of Emergency Medicine, Lo-Hsu Medical Foundation Lotung Poh-Ai Hospital, Yilan, Taiwan; 5 School of Nursing, National Yang Ming Chiao Tung University, Taipei, Taiwan; Universita Politecnica delle Marche, ITALY

## Abstract

**Aims:**

The number of ventilator-dependent patients is rapidly increasing globally. As a result, long-term mechanical ventilation (LTMV) patients face the choice of receiving health care in respiratory care wards (RCWs) rather than at home. In this study, we applied Andersen’s behavioral theoretical model (ABM) to investigate the determinants of the health care service decisions of patients receiving LTMV.

**Methods:**

A cross-sectional research design and cluster random sampling were used to select 365 participants from nine RCWs and eight home care facilities in northern Taiwan. Data were collected in face-to-face interviews using a structured questionnaire.

**Results:**

Of the predisposing factors, advanced age and an education level of at least junior high school influenced the choice to use RCW services. Being married, living with extended family, and medium or higher socioeconomic status were associated with the decision to use home care services. Of the enabling factors, patients with more caregivers, those whose family caregivers held strong beliefs about providing care, and those who perceived greater social support from health care providers were more likely to choose home care services. Of the need factors examined, poor cognitive function and higher dependence on assistance for activities of daily living (ADL) increased the probability of patients choosing RCW services. Hierarchical logistic regression analysis indicated that our final model accounted for 44.8% of the observed variance in health care service choice.

**Conclusions:**

ABM enables an improved understanding of the health care service choices of LTMV patients. Our findings also highlight the importance of rigorously assessing patient needs and helping patients choose the most appropriate health care service.

## Introduction

Currently, most critically ill patients with COVID-19 are receiving mechanical ventilation (MV) worldwide. Studies have estimated that 6%– 37% of patients who require MV in intensive care units (ICUs) eventually require long-term mechanical ventilation (LTMV) [1, 2]. Therefore, the number of ventilator-dependent patients is rapidly increasing worldwide. The most widely used definition of LTMV is the continuous use of MV for at least six hours per day over a period of more than 21 days [3].

The prevalence of LTMV varies among countries. In Europe, Canada, and the US, 7–15 people per 100,000 require LTMV [3–6]. In Taiwan, the rate is considerably higher, at 26 people per 100,000 [7]. To more effectively manage ventilator-dependent patients and control medical expenses for LTMV [8], the National Health Insurance Administration (NHIA) of Taiwan has established an integrated delivery system (IDS) with a comprehensive prospective payment program for LTMV patients [8]. This system comprises integrated payments with a managed care system that covers mechanical ventilators in four phases of care and payment schemes: a fee-for-services payment in ICUs covers patients who require acute respiratory care who stay in the ICU for up to 21 days. After 42 days of per-diem payment, if MV is still needed, patients are transferred to a respiratory care center (RCC) or to subacute care for aggressive weaning of ventilator support for LTMV patients. After 64 days of unsuccessful liberation from MV, patients who become ventilator-dependent are transferred to a per-diem respiratory care ward (RCW) and/or per-diem home care services [8, 9]. An RCW provides 24-hour skilled nursing for ventilator-dependent patients who need long-term care. The home care services support patients who are cared for directly by family caregivers at home by providing additional care from a multidisciplinary team [10].

To facilitate improved home care services, it is crucial to implement policies that shift resources toward community-based care for LTMV patients. Currently, most such patients and their families show a preference for RCW institutional care services. According to statistics published in 2011 by the Taiwanese Ministry of Health and Welfare, only 8.1% of LTMV patients select home care services after being discharged from the hospital, in contrast to patients in Canada and the US, of whom 11.0–30.8% choose home care services [11, 12].

To investigate the factors determining health care service choice, Gehlbach and Salamanca [13] conducted a cohort study involving 548 MV patients in the ICU. The researchers found that patient age, duration of MV use, cognitive dysfunction, and functional status were all associated with the decision to be discharged from a care facility. There is a large body of research that examines the role of patient-related [13] economic and environmental factors [14]. However, the role of resource factors in determining health care service choices for LTMV patients is less well understood. Andersen’s Behavioral Model (ABM) proposes that predisposing, enabling, and need factors [15] have been widely used to explore the determinant factors of health care service use. There is little research using ABM as a theoretical framework for exploring the determinants of LTMV patients’ health care choices. The present study seeks to fill this gap in the literature by using the ABM to examine influencing factors of health care service choices among LTMV patients.

## Methods

### Conceptual framework: Andersen’s behavioral model (ABM) of health service use

The study applied an ABM theoretical model to identify the factors associated with health care service use. Therefore, the study hypothesized that predisposing, enabling and need factors significantly influence the health service choices of LTMV patients.

#### Predisposing factors

Predisposing factors characterize the propensity of an individual to use health care services, which include demographic characteristics and social structure variables [16]. Social structure variables comprise a broad array of factors that reflect the location of the individual within society and the available health care resources [17], which include family structure and socioeconomic status (SES). Family structure and SES variables both exert a strong influence on whether an individual chooses health care services [18, 19].

#### Enabling factors

Enabling factors are defined as the resources available to an individual/society/community that allow the use of health care services [16]. Individuals with more resources, such as family household income and the number of caregivers available, affect long-term health care service utilization [18]. Family caregivers, as key resources in providing home care, may be more likely to maintain and reinforce caregiving behaviors [20]. In Chinese culture, the beliefs of family caregivers may also determine a patient’s health care service choices and be an enabling factor. Social resources that include government subsidies and social support variables may influence the healthcare service choices of LTMV [21]. Sources of social support can promote a patient’s physical and psychological adaptation, including social networks, and may have an impact on health care service use [22]. These social networks extend beyond immediate family to encompass health care providers such as nurses, physicians, and respiratory therapists; we treat the support arising from family and from health care providers as two separate variables. The degree of urbanization in the area in which a patient lives provides a measure of community resources [18]. Individuals living in more urbanized areas may have greater access to health care resources, which may positively influence the availability of health care services [23].

#### Need factors

Need factors refer to the self-perceived needs of an individual given his or her general health and functional status and may influence the individual’s decision to access care [24]. Individual perceived needs can be influenced positively or negatively by the patient’s number of chronic conditions, duration of ventilator use, daily function status [25, 26] and cognitive function [22, 26], which have been associated with health care service choices.

Based on the ABM, we developed a framework and hypothesized that a range of predisposing, enabling, and need factors influence LTMV patients and their family members when deciding to use RCW or home care services.

### Participants and sampling

In Taiwan, the NHIA is administered by the Ministry of Health and Welfare according to six divisions: the Taipei, Northern, Central, Southern, Kaoping, and Eastern regions. The Taipei regional division is the largest in the country and comprises four counties: Taipei City, New Taipei City, Keelung City, and Yilan County (excluding Kinmen and Lienchiang). This region accounts for 38.7% of all LTMV patients in Taiwan, which is the largest proportion accounted for by any region. We therefore used LTMV patients within this regional division as our study population.

All LTMV patients are required to join IDS and are entitled to receive care with full financial coverage either in RCWs or home care services. Patients and family members (surrogates) in conjunction with the physician’s professional judgment and their expectations and preferences make the decision to transfer patients to an RCW or home care service and requires patient and or family member (surrogates) agreement. The criteria for admission to the RCW were as follows: 1) ventilator use for more than 63 days and unsuccessful liberation from MV; 2) stable medical conditions, such as hemodynamic and respiratory status with stable ventilation. The cost of home care services for LTMV are reimbursed by the NHIA, which requires requests to be submitted and get it approved by IDS for home care facilities (also called home care agencies or home care units in affirmed hospitals) and meet the following criteria: (a) patients must need respiratory services teams, including physicians, respiratory therapists, nurses, and case managers. (b) The nurses and respiratory therapists must provide home visits twice a month and physician visits once every two months for at least an hour.

We obtained a list of RCWs and home care facilities from the NHIA website and found a total of 44 RCWs and 22 home care facilities in the Taipei region that were eligible for inclusion in the study.

The sample size was determined using the Katz [27] rule. In multiple logistic regression, the sample size should allow for at least 20 observations per variable. This study included 17 variables, dictating a minimum sample size of 340. After determining the proportion of facilities in each county, we conducted a cross-sectional study with cluster random sampling to select nine RCW facilities and eight home care facilities from which to draw our samples. The eligibility criteria include that the patient admitted to the RCW or home care facility in stable condition, that no active ventilator weaning plan was in place, and that the patient does not require transfer to an RCC or ICU setting.

### Data collection

Data were collected via face-to-face interviews from November 2016 to June 2017. This study was approved by the institutional review boards of an affiliated hospital (approval number: TH-IRB-0016-0031) and a Taiwan university-affiliated hospital (approval number: 2016A022). The researchers explained the purpose of the study to each facility, and interested individuals were invited to participate. Written informed consent was obtained from each participant (patients and their primary family member) after the study purpose was explained. Arrangements were made for visits to RCW facilities and patients’ homes. Interviews were conducted in private rooms in the hospital wards and homes whenever possible. All questionnaire responses were anonymized by assigning unique numbers to represent each participant. Four interviewers were trained by the researchers, and a six-patient intraclass correlation coefficient was calculated. An interrater reliability score of 0.92 was obtained, indicating a high degree of reliability among the four interviewers [28]. Finally, 367 participants were interviewed, of whom 365 completed the survey questionnaires for an overall response rate of 99.5%.

### Dependent variable

The health care service choice made by the LTMV patient was the dependent variable in the study. It was coded as a binary variable, with a value of 0 and 1 when the patient received care in an RWC and home care services, respectively.

### Independent variables

The independent variables included in the model were predisposing, enabling and need factors as follows:

Of the predisposing factors, the variables were age, sex (male, female), marital status (single, non-single), education level (illiterate, elementary school, junior high school, senior high school, college education), family structure [living alone, living in a nuclear family (i.e., parents with children), living with two or more generations as an extended family], and SES.

The Two-Factor Index of Social Position Questionnaire was used to measure SES, specifically the position of the patient within the societal structure [29]. We used a version modified by Lin [30] that employs a composite measure that is directly determined by the education and occupation status. The obtained scores ranged 11–55 and were categorized as high (41–55), intermediate (30–40), or low (11–29) SES levels.

Of the enabling factors, the variables were the number of caregivers, beliefs held by the family caregivers relating to care, social support offered by family, social support offered by health care providers, annual family household income, degree of urbanization, and provision of government subsidies (binary: yes, no).

The Family Caregiver Belief Scale (FCBS) was used to assess the beliefs of family caregivers. We used the Chinese version of the FCBS (C-FCBS) that was developed by Hsu [31]. It contains 18 items divided into three domains: value of care (seven items), utility of reciprocal care (five items), and commitment to care (six items). Each item was scored on a five-point Likert scale (1 = strongly disagree to 5 = strongly agree). Total scores were 18–90 points, with higher scores indicating stronger caregiver beliefs regarding the provision of care.

A Social Support Questionnaire (SSQ) [32] was used to measure the levels of support the patient perceived that they received from their family members and health care providers. The SSQ contains 16 items divided into four domains: emotional support (four items), informational support (four items), appraisal support (four items), and instrumental support (four items). Each item was scored on a four-point Likert scale (0 = almost never to 3 = almost always). Total scores ranged from 0–48 points; higher scores indicated a higher perceived level of support from family members and health care providers.

The level of urbanization was measured according to the township classification by Liu, Hung [33]. All 365 townships in Taiwan can be classified into seven categories ranging from rural to highly urbanized.

Regarding the need factors, the included variables were the number of chronic diseases, duration of ventilation, cognitive function, and daily living functioning.

The Short Portable Mental State Questionnaire (SPMSQ) was used to measure the cognitive function of the participants. The SPMSQ uses a 10-item cognitive screening scale developed by Pfeiffer [34] and is scored with a 0 for a correct response and a 1 for an incorrect response. Thus, the participant’s score reflects the number of incorrect responses. Total scores range from 0–10 points and are categorized into three levels of cognitive function: < 3 errors, indicating normal cognitive function; 3–4 errors, indicating mild cognitive impairment; and > 5 errors, indicating moderate to severe cognitive impairment.

The Barthel index developed by Mahoney and Barthel [35] was used to assess the ability of LTMV patients to carry out basic personal care functions in their everyday lives. The scales are categorized into ten areas: eating, positioning, grooming, toileting, bathing, ambulating, dressing, transferring, bowel continence, and bladder continence. Total scores ranged 0–100 points and are categorized into four levels of daily living function: 0–20 (total dependence), 21–60 (severe dependence), 61–90 (moderate dependence), and 91–100 (mild dependence or independence).

### Data analysis

Data analysis was performed using SPSS 22.0 software (Chicago, Ill). We used frequency, percentage, mean, and standard deviation (SD) and performed independent t tests or chi-squared tests to examine the distribution of the data and identify relationships among the three types of factors (predisposing, enabling, and need) and health care service type use. Because of the categorical nature of the dependent variables, a hierarchical logistic regression was performed, and the candidate explanatory factors were incorporated into the model as dependent variables.

## Results

The age of the study population was 66.67 ± 20.41 (mean ± SD) years, and slightly more than half of the participants were male (53.4%) and non-single (56.4%) (Table 1). Nearly one-third (30.7%) had an elementary school-level education only, and 69.6% were members of a nuclear family. Finally, 52.3% had low SES. The patients had 1.91 ± 0.92 family caregivers available to provide care (Table 1). These caregivers demonstrated a moderately strong level of belief (3.49 ± 0.61) in providing care to the patients, and the patients perceived a moderate degree of social support from both their health care providers (2.22 ± 0.64) and their family members (2.20 ± 0.67). Almost half of the patients resided in highly urbanized areas (46.8%), and their annual household income was USD 27,681.7 ± 28,159.5. Over three-quarters (78.9%) of the patients did not receive government subsidies. The patients exhibited 1.98 ± 1.21 chronic diseases, and the duration of ventilator use was 3.56 ± 2.51 years (Table 1). Over half of the patients (56.4%) had moderate to severe cognitive impairment. Finally, a large proportion (80.5%) were severely or totally dependent on assistance for activities of daily living (ADL).

**Table 1:** Characteristics of long-term mechanical ventilation patients (N = 365).

Variables	Total		Home care		Institutional care		*χ* ^*2*^ */t*	*p*
	(N = 365)		(n = 168)		(n = 197)			
	n	%	n	%	n	%		
**Predisposing factors**								
Age (M, SD)	66.67	20.41	61.94	19.50	70.71	20.05	4.18	0.804
Sex							0.03	0.874
Male	195	53.4	89	53.0	106	53.8		
Female	170	46.6	79	47.0	91	46.2		
Marital status							3.78	0.047
Single	159	43.6	64	38.1	95	48.2		
Non-single	206	56.4	104	61.9	102	51.8		
Education level							2.79	0.594
Illiterate	67	18.4	26	15.5	41	20.8		
Elementary school	112	30.7	54	32.1	58	29.4		
Junior high school	59	16.2	25	14.9	34	17.3		
Senior high school	67	18.4	32	19.0	35	17.8		
College and more	60	16.4	31	18.5	29	14.7		
Family structure							12.25	0.002
Living alone	40	11.0	8	4.8	32	16.2		
Living with nuclear family	254	69.6	125	74.4	129	65.5		
Living with extended family	71	19.5	35	20.8	36	18.3		
Socioeconomic status (SES; M, SD)	29.30	11.14					0.66	0.719
High SES	64	17.5	32	19.0	32	16.2		
Intermediate SES	110	30.1	48	28.6	62	31.5		
Low SES	191	52.3	88	52.4	103	52.3		
**Enabling factors**								
Number of caregivers	1.91	0.92	1.71	0.76	2.08	1.01	3.96	< 0.001
Family caregiving beliefs (M, SD)	3.49	0.61	3.74	0.59	3.27	0.52	-7.92	< 0.001
Social support (health care providers) (M, SD)	2.22	0.64	2.33	0.57	2.12	0.67	-3.13	0.002
Social support (family) (M, SD)	2.20	0.67	2.32	0.57	2.10	0.74	-3.23	0.001
Level of urbanization							1.60	0.449
Rural/general urban areas	85	23.3	43	25.6	42	21.3		
Moderately urban areas	109	29.9	52	31.0	57	28.9		
Highly urban areas	171	46.8	73	43.5	98	49.7		
Household years income (USD)^†^	27681.7	28159.5	30369.2	37630.8	25389.8	15956.6	-1.59	0.112
Subsidies							0.87	0.351
Yes	70	19.2	28	17.4	42	21.3		
No	288	78.9	133	82.6	155	78.7		
**Need factors**								
Number of chronic diseases (M, SD)	1.98	1.21	1.86	1.16	2.08	1.24	1.68	0.093
Ventilator use in years (M, SD)	3.56	2.51	3.85	2.26	3.32	2.68	-1.98	0.048
Cognitive function (M, SD)	2.20	0.94					95.53	< 0.001
Normal	133	36.4	105	62.5	28	14.2		
Mildly impaired	26	7.1	12	7.1	14	7.1		
Moderate/severely impaired	206	56.4	51	30.4	155	78.7		
Barthel index (M, SD)	23.21	34.48					88.25	< 0.001
Mild/independent	33	9.0	33	19.6	0	0		
Moderately dependent	38	10.4	35	20.8	3	1.5		
Severely/totally dependent	294	80.5	100	59.5	194	98.5		

M, mean; SD, standard deviation;

^†^1 USD = 31 NTD;

Regarding LTMV service choice, a total of 197 patients (54.0%) had chosen to utilize the RCW service, with the remaining 168 (46.0%) receiving home care services (Table 1). According to the bivariate analysis of the predisposing factors, we found that patients who were non-single (*t* = 3.78, *p* = 0.047) and lived with extended family (χ^2^ = 12.25, *p* = 0.002) were significantly more likely to choose home care services.

Regarding enabling factors, we found that stronger caregiving beliefs among family caregivers (*t* = −7.92, *p* < 0.001) and greater social support from health care providers (*t* = −3.13, *p* = 0.002) and family (*t* = −3.23, *p* = 0.001) were significantly positively associated with the selection of home care service. However, patients with more caregivers (*t* = 3.96, *p* < 0.001) were more likely to choose RCW services. In terms of need factors, we found that fewer years of ventilator use (*t* = −1.98, *p* = 0.048), moderate to severe cognitive impairment (χ^2^ = 95.53, *p* < 0.001), and severe to total dependence on assistance for daily functions (χ^2^ = 88.25, *p* < 0.001) were positively associated with the use of RCW services. Regarding the factors that affect health care service choice, in the first hierarchical logistic regression model (Model 1) for the factors influencing the selection of RCW or home care service, which included only predisposing factors, we found that older patients (odds ratio [OR] = 0.97, 95% confidence interval [CI] **=** 0.96–0.98) were more likely to choose RCW services, while patients who were non-single (OR = 1.85, 95% CI **=** 1.11–3.86) chose home care services 1.85 times more often than single patients. Patients living with their nuclear (OR = 2.54, 95% CI **=** 1.06–6.06) or extended families (OR = 3.01, 95% CI **=** 1.15–7.90) were more likely to choose home care services than patients who lived alone (Table 2).

**Table 2:** Hierarchical logistic regressions of utilization among patients with long-term ventilator dependence.

Items	Model 1			Model 2			Model 3		
	β	OR (95% CI)	p value	β	OR (95% CI)	p value	β	OR (95% CI)	p value
**Predisposing factors**									
Age	-0.03	0.97 [0.96, 0.98]	**< 0.001**	-0.03	0.97 [0.96, 0.98]	**< 0.001**	-0.02	0.98 [0.96, 0.99]	**0.039**
Sex (reference: male)	0.14	1.15 [0.72, 1.84]	.550	0.17	1.81 [0.68, 2.04]	0.551	-0.01	0.98 [0.52, 1.88]	0.969
Marital status (reference: single)									
Non-single	0.62	1.85 [1.11, 3.86]	**0.018**	0.71	2.03 [1.46, 3.59]	**0.015**	0.73	2.08 [1.01, 4.27]	**0.046**
Education (reference: illiterate)									
Elementary school	0.38	1.47 [0.73, 2.93]	0.365	0.01	1.01 [0.46, 2.20]	0.983	-0.52	0.59 [0.23, 1.51]	0.983
Junior school and more	-0.09	0.91 [0.46, 1.80]	0.787	-0.52	0.59 [0.27, 1.28]	0.119	-0.99	0.37 [0.15, 0.91]	**0.031**
Family structure (reference: living alone)									
Living with nuclear family	0.93	2.54 [1.06, 6.06]	**0.036**	0.65	1.92 [0.69, 5.29]	0.207	1.34	3.81 [0.90, 16.09]	0.068
Living with extended family	1.10	3.01 [1.15, 7.90]	**0.025**	1.06	2.89 [0.94, 8.89]	0.064	2.16	8.63 [1.80, 41.34]	**0.007**
SES (reference: low SES)									
Intermediate SES	0.11	1.12 [0.66, 1.89]	0.675	0.23	1.26 [0.69, 2.30]	0.450	0.76	2.13 [1.01, 4.49]	**0.048**
High SES	0.47	1.60 [0.85, 3.01]	0.143	0.49	1.64 [0.75, 3.58]	0.219	1.28	3.59 [1.40, 9.18]	**0.008**
**Enabling factors**									
Number of caregivers				-0.43	0.65 [0.48, 0.87]	**0.004**	-0.38	0.68 [0.47, 0.97]	**0.034**
Family caregiving beliefs				0.08	1.09 [1.06, 1.12]	**< 0.001**	0.07	1.07 [1.04, 1.11]	**< 0.001**
Social support (family)				-0.06	0.94 [0.59, 1.49]	0.795	-0.17	0.83 [0.47, 1.48]	0.544
Social support (health care providers)				0.52	1.68 [1.05, 2.66]	**0.029**	0.57	1.44 [1.03, 2.48]	**0.039**
Family yearly income (USD)				0.00	1.00 [1.00, 1.00]	0.231	0.00	1.00 [1.00, 1.00]	0.340
Level of urbanization (reference: general)									
Moderately urban areas				-0.08	0.92 [0.46, 1.85]	0.818	-0.05	0.95 [0.39, 2.28]	0.915
Highly urban areas				-0.46	0.63 [0.33, 1.22]	0.169	-0.46	0.63 [0.28, 1.41]	0.261
Subsidies (reference: no)									
Yes				0.37	1.45 [0.75, 2.82]	0.266	0.46	1.58 [0.72, 3.48]	0.253
**Need factors**									
Number of chronic diseases							-0.09	0.91 [0.69, 1.20]	0.529
Ventilator use, years							0.07	1.07 [0.95, 1.21]	0.237
Cognitive function (reference: moderate)									
Mild impairment							1.29	3.64 [1.26,10.58]	**0.017**
Normal							2.06	7.87 [3.72,16.64]	**< 0.001**
Barthel index (reference: severely dependent)									
Moderately dependent							2.03	7.57 [1.84,31.27]	**0.005**
Mildly dependent and independent							20.58	874.4 [0.00, NA]	0.997
**-2 log likelihood**		455.00			384.12			279.63	
**Cox-Snell R** ^**2**^		**0.100**			**0.262**			**0.448**	
**Hosmer–Lemeshow test**		*χ* ^*2*^	0.228		χ^2^	0.485		*χ* ^*2*^	0.081
		10.562			7.486			14.021	

SES, socioeconomic status

In Model 2, which included both the predisposing and enabling factors, we found that only age and marital status remained significant. In addition, patients with higher numbers of family caregivers (OR **=** 0.65, 95% CI = 0.48–0.87) were more likely to choose RCW services, and those with caregivers who held stronger caregiving beliefs (OR **=** 1.09, 95% CI = 1.06–1.12) tended to choose home care services. A greater level of perceived social support from health care providers (OR **=** 1.68, 95% CI = 1.05–2.66) was also more likely to lead to the selection of home care services.

In Model 3, all three factors (predisposing, enabling, and need) were included (Table 2). Of the predisposing factors, we found that older patients were more likely to choose RCW services, and patients who had completed at least junior high school were more likely than illiterate patients to choose RCW services. By contrast, non-single patients and those residing with extended family were more likely to choose home care services than patients who lived alone. Patients with a high or medium SES were more likely to choose home care services than patients with a low SES.

Regarding the enabling factors, the same variables that were included in Model 2 were included in Model 3. Patients with higher numbers of family caregivers were again more likely to choose RCW services, whereas those whose family caregivers held stronger caregiving beliefs and those who felt they received more social support from their health care providers were more likely to choose home care services. Of the need factors, we included the cognitive function and daily functions in Model 3. Patients with normal cognitive function (OR **=** 7.87, 95% CI **=** 3.72–16.64) or mild cognitive impairment (OR **=** 3.64, 95% CI **=** 1.62–10.58) were more likely to choose home care services than those with moderate cognitive impairment. Patients with a moderate level of dependence for daily functions (OR **=** 7.57, 95% CI **=** 1.84–31.27) were more likely to choose home care services than those with severe dependence. We calculated the Cox-Snell *R*^2^ value for Model 3 and found that the model accounted for 44.8% of the variance in long-term health care service utilization (Table 2).

## Discussion

We used the ABM to identify factors that affect health care service utilization by LTMV patients. The model explained 44.8% of the variance in their choice of long-term health care service. In our sample, 54.0% of LTMV patients chose to reside in RCWs, while the remaining 46.0% relied on home care services. This was similar to the findings of a cohort study by Hill and Fowler [2], in which 43.5% of the patients selected home care services; however, our study indicated a lower percentage of choosing home care services than that of reported by Gehlbach and Salamanca [13], which was 73.9%. One possible explanation could be related to out-of-pocket health expenditures. In Taiwan, the health insurance fully covers LTMV patients’ health care services at RCWs or home care services, patients or their families only pay low out-of-pocket costs. In the United States, if individuals decide to use institutional services, the amount of services is decided by health providers [36] results in higher out-of-pocket expenditures than home care services [37]. Hence, there appears to be a higher probability of patients selecting home care services.

We found that older LTMV patients were more likely to choose an institutional care model. This finding is similar to results elsewhere in the Gehlbach and Salamanca study [13]. A possible explanation is that older patients have more severe illnesses and chronic conditions [13, 38] and may therefore be in greater need of professional health care services and may rely more heavily upon institutional care. Patients with higher education levels were more likely to choose institutional services. Our findings are similar to those of Tzeng [39], who indicated that patients with higher education were more likely to choose institutional care. However, this finding contrasts with those of Wu and Hu [25] and Kim and Cho [26], who found that education level was not associated with long-term health care utilization. The reason for these differences may be the distribution of education levels within the study samples. In our study, 51.0% of the participants had completed junior high school and therefore had received at least seven years of education. By contrast, in Wu and Hu [25] sample, only 22.8% of the long-term elderly patients in their sample had completed junior high school, while only 38.4% of the stroke patients in the study by Kim and Cho [26] had more than six years of education. A higher level of education is associated with a higher SES, probably because of which a more educated patient is more likely to choose professional or institutional health care services [40].

Regarding family structure, patients living with extended family were more likely to choose home care services. Our findings are supported by Zhang and Zeng [40], who reported that patients living with a spouse, children, and extended family members may enjoy greater access to family resources [40]. Elsewhere, evidence suggests that residing with extended family engenders a greater number of relationships with other family members and stronger attitudes of thoughtfulness among members [41]. It is reasonable to expect patients living with extended family to be more likely to choose home care services.

Differences in the SES may reflect differences in social class, and an individual with a higher SES is assumed to have a greater sense of mastery and control and may be more likely to select the best health care service available [42]. High-SES families have more resources available with which to facilitate a patient’s transition from institutional care to home care. In addition, caregivers may exhibit greater role readiness and adaptability in high-SES families than in low-SES families [42]. Thus, it is understandable that a higher SES is a determinant of the selection of home care services among LTMV patients. Our findings regarding sex are concordant with those obtained elsewhere, which suggest that sex does not significantly affect health care service choice [13, 25, 26, 38, 40].

Of the enabling factors investigated, patients with more caregivers were more likely to choose RCW services. This finding is similar to that of Lee [43] but dissimilar from those of a number of other studies [25, 40, 41]. One possible explanation may be related to the severity levels of disability. LTMV patients with a higher severity level of disability require 24-hour daily care and total assistance and caregivers competent in skills, such as nasogastric feeding, inhalation, suction, oxygen cannula, and saturation monitoring, as well as in ventilator techniques. In the present study, of the LTMV patients who received RCW services, 98.5% had severe to total dependence for daily functions, higher than in case of those who received home care services (59.5%). In addition, among the home care patients in this study, 80 (47.6%) of their families hired people for additional assistance for patient care and life assistance at home, often facilitating patients receive 24-hour care for patients [44]. However, significant difference was observed in the availability of the number of family caregivers between RCW and home care services. Thus, taking into consideration the availability of additional people in home care services, it may not be sufficient to differentiate between use of RCW and home care services. Future studies should examine the relationship of the number of caregivers and their competence with health care service choice for LTMV patients.

This is the first study to examine the relationship between family caregiver beliefs surrounding the provision of care and the choice of health care service. We found that the beliefs of family caregivers influenced the health care service choices of LTMV patients. The measures used in this study reflect the attitudes of the primary caregiver toward the patient [45]. Family caregivers with stronger beliefs about the importance of providing care are likely to exhibit a stronger commitment to patient care [46, 47]. Such caregiver beliefs can therefore motivate patients to choose to receive health care at home rather than in an RCW setting [48]. Therefore, stronger family caregiver beliefs in the importance of providing care are potentially linked to a stronger commitment to caregiving by family members and may in turn make patients more likely to choose home care services.

The social support offered by health care providers was identified as a significant factor determining health care service choice. When LTMV patients require relocation or a change in health care service type, professional advice and relevant resources can greatly reduce their psychological distress and that of their family members if there are conflicts involved in this change [49, 50]. Furthermore, support from health care providers helps patients and family members cope with the realities of receiving and providing care at home [50]. Greater levels of support from health care providers can also facilitate adaptation to both the patient and caregiver roles [50, 51], and formal support from health care providers can facilitate improved adherence among patients and promote effective health care behaviors [51, 52]. Therefore, when patients perceive greater levels of social support from health care providers, they are understandably more willing to choose home care services.

Social support from the family was not found to be associated with health care service choice. One possible explanation for this finding relates to psychological burden. In a study by Liu and Lu [53], the majority of family caregivers of LTMV patients experienced a heavy psychological burden, regardless of whether the patient was receiving institutional care or home care. This burden may explain the lower levels of social support offered by family members than by health care providers.

Another enabling factor, urbanization, did not significantly affect health care service choice. This finding is similar to that of Pai and Kung [14] but dissimilar to findings of Wu and Hu [25] and Zhang and Zeng [40]. These differences in research findings may be attributable to differences in the study setting. Our study uses a sample drawn from North Taiwan, a region where resources are allocated relatively uniformly across areas; therefore, the level of urbanization may not have varied sufficiently to significantly affect health care service choice. Household income also did not exert a significant effect on health care service choice, which is similar to the findings of Luppa and Luck [22] and Wu and Hu [25]. The receipt of government subsidies also exerted no significant effect. In Taiwan, the government provides welfare subsidies for long-term care equipment, such as respiratory therapy equipment, wheelchairs, and pressure air mattresses. All LMTV patients and family members who receive welfare subsidies select either RCW or home care services with equally likelihood. Therefore, government subsidies may not significantly affect health care service choices.

Regarding need factors, we found that cognitive function and ADL had significant effects on health care service choice. Not surprisingly, LTMV patients with normal or mild cognitive impairment were 7.87 and 3.64 times more likely to choose home care services, respectively. Our findings are similar to those obtained by other studies [13, 22, 26]. Cognitive impairment is an aging-related disease [54], and elderly patients with more chronic diseases also tend to exhibit more cognitive function decline [13, 38]. Severe cognitive impairment is a strong influencing factor of institutional care choice. The degree of disability also has a significant influence on patients’ institutional care choice. Our findings are similar to those obtained by other researchers [13, 22, 26, 40]. Patients with more severe cognitive impairment are more limited in their capacity to perform ADL and therefore require more support and assistance. Thus, older patients who are highly dependent on assistance for ADL are more likely to choose institutional care.

Neither the number of chronic diseases nor the number of years of ventilator use significantly affected health care service choice. Our findings are supported by results obtained by other researchers [13, 55]. It is worth noting, however, that the within-sample variation in the number of chronic diseases and the duration of ventilator use in this study were low, which limited the ability of the regression analysis to identify the relationship between these variables and health care service choice.

A limitation of our study is the use of a retrospective cross-sectional analysis design for enrolled LTMV patients receiving home or institutional care. In reality, the choice of health care service for LTMV patients may be a dynamic process. Therefore, capturing a longer-term picture of the health care service choices of such patients is likely to provide a more comprehensive understanding. In the future, we recommend that a longitudinal prospective study be conducted to obtain a more accurate understanding of the influencing factors of health care service choices of LTMV patients, specifically how such choices evolve over time. In addition, based on our finding that family caregivers play an important role in the health care service choices of LTMV patients, we recommend that future studies focus more on caregiver-related factors, such as caregiver burden.

## Conclusion

To our knowledge, the present study is the first to use the ABM to identify factors that affect health care service choices among LTMV patients. Our model offers a number of valuable insights into the determinants of the health care service choice of LTMV patients from both theoretical and practical perspectives. Our model accounts for 44.8% of the observed variance in health care service choice. Evidently, there is more to the question of health care service choice that has yet to be explored. Our study indicates that patients who are older and have higher education levels, more caregivers, more severe cognitive impairment, and a higher level of dependence are more likely to choose RCW services. By contrast, patients who are not single, reside with extended family, receive more social support from health care providers, have a higher SES, and have caregivers who express stronger beliefs in the importance of providing care are more likely to choose home care services. It is therefore imperative that more efforts be made to reinforce primary caregivers’ beliefs in the importance of providing care. It is also important to increase the level of social support offered by health care providers so that patients and their family members are better positioned to choose the most appropriate health care service. Future research should explore the cost-effectiveness or cost utility of different health care service models.

## Supporting information

S1 File (PDF)
